# PGA: an R/Bioconductor package for identification of novel peptides using a customized database derived from RNA-Seq

**DOI:** 10.1186/s12859-016-1133-3

**Published:** 2016-06-17

**Authors:** Bo Wen, Shaohang Xu, Ruo Zhou, Bing Zhang, Xiaojing Wang, Xin Liu, Xun Xu, Siqi Liu

**Affiliations:** BGI-Shenzhen, Shenzhen, 518083 China; Beijing Institute of Genomics, Chinese Academy of Sciences, Beijing, 100101 China; Department of Biomedical Informatics, Vanderbilt University School of Medicine, Nashville, TN 37232 USA

**Keywords:** Proteomics, RNA-Seq, MS/MS, Peptide identification, Proteogenomics

## Abstract

**Background:**

Peptide identification based upon mass spectrometry (MS) is generally achieved by comparison of the experimental mass spectra with the theoretically digested peptides derived from a reference protein database. Obviously, this strategy could not identify peptide and protein sequences that are absent from a reference database. A customized protein database on the basis of RNA-Seq data is thus proposed to assist with and improve the identification of novel peptides. Correspondingly, development of a comprehensive pipeline, which provides an end-to-end solution for novel peptide detection with the customized protein database, is necessary.

**Results:**

A pipeline with an R package, assigned as a PGA utility, was developed that enables automated treatment to the tandem mass spectrometry (MS/MS) data acquired from different MS platforms and construction of customized protein databases based on RNA-Seq data with or without a reference genome guide. Hence, PGA can identify novel peptides and generate an HTML-based report with a visualized interface. On the basis of a published dataset, PGA was employed to identify peptides, resulting in 636 novel peptides, including 510 single amino acid polymorphism (SAP) peptides, 2 INDEL peptides, 49 splice junction peptides, and 75 novel transcript-derived peptides. The software is freely available from http://bioconductor.org/packages/PGA/, and the example reports are available at http://wenbostar.github.io/PGA/.

**Conclusions:**

The pipeline of PGA, aimed at being platform-independent and easy-to-use, was successfully developed and shown to be capable of identifying novel peptides by searching the customized protein database derived from RNA-Seq data.

**Electronic supplementary material:**

The online version of this article (doi:10.1186/s12859-016-1133-3) contains supplementary material, which is available to authorized users.

## Background

Using tandem mass spectrometry (MS/MS) data, database-dependent searching is a popular approach for peptide identification. The searching relies on the completeness and quality of the reference database of the proteome. If a correspondent peptide sequence is not listed in the reference database, an MS/MS spectrum, even at high quality, would fail to identify a peptide. Generation of a comprehensive reference database is therefore a challenging task in bioinformatics analysis towards MS/MS signals. Some common databases, such as Ensembl [1], RefSeq [2], and UniProt [3], cannot satisfactorily meet this urgent requirement; however, some new solutions have recently been proposed to improve the completeness of proteome databases. Through some attempts, such as six-frame translation from the genome [4] and expressed sequence tags (ESTs) [5], including known coding variations [6] and alternative splicing events [7], databases with such combined information were constructed to offer opportunities to expand the data body of novel splices, genomic variants, and new genes. However, these methods lead to significantly increased database sizes but do not greatly improve the sensitivity of peptide identification. Recent studies have reported advances in peptide or protein identification with the aid of transcriptome databases, which were obtained from the unprecedented capabilities of high-throughput next-generation sequencing [8–12]. RNA-Seq technology indeed has provided qualitative or quantitative gene expression information on a whole genome scale at a single-base resolution. Since transcriptomic and proteomic analyses could be done on the same cells or tissues, a sample-specific database based upon RNA-Seq data would significantly enhance sensitivity for peptide identification and improve accuracy for finding novel peptides. Importantly, for non-model species whose genome sequences are absent, the transcript sequences derived from RNA-Seq data by *de novo* transcriptome assembly would be beneficial to construct the proteomic database for MS/MS searching. In this strategy, the technique bottleneck is how to create an accessible and flexible bioinformatic pipeline that efficiently harnesses RNA-Seq data for the discovery of protein variations [13]. According to our knowledge, three new software, customProDB, an R package developed by Wang et al. [14], a workflow within Galaxy-P generated by Sheynkman et al. [13], and sapFinder developed by Wen et al. [9], have made important contributions to this field. However, customProDB only provides functions for database construction without offering functions for downstream analysis, such as database searching and post-processing, which are also very important for novel peptide identification. Galaxy-P provides functions for the SAP database and splice database; however, it does not include a function for novel transcript-coded peptides. The software sapFinder mainly focuses on the peptides related to single amino acid polymorphisms but not for general detection of novel peptides. Therefore, there is still much room for improved identification of novel peptides through the construction of a comprehensively customized proteomics database based upon RNA-Seq data.

Herein, we describe PGA, an R/Bioconductor package which enables an automatic process for constructing customized proteomic databases based upon RNA-Seq data with or without guidance from a reference genome, searching peptides using MS/MS data, post-processing and generating an HTML-based report with a visualized interface.

## Implementation

**Fig. 1:**
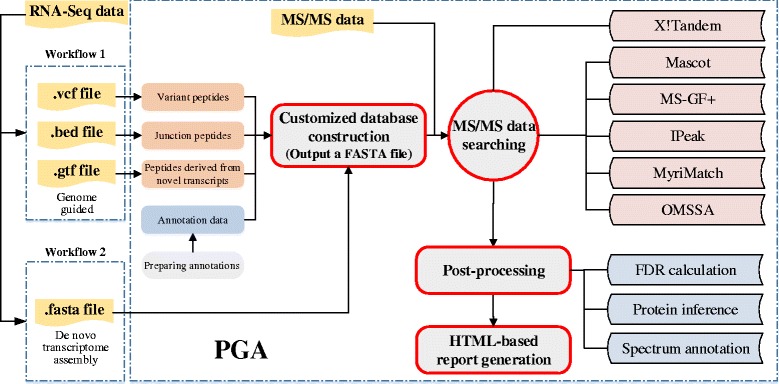
Schematic overview of PGA package

As illustrated in Fig. 1, the workflow for identification of novel peptides using the customized database derived from RNA-Seq data is broadly divided into four steps as below.

### Construction of the customized proteomic database

There are two kinds of customized proteomic databases created with PGA. One was constructed from the analysis of RNA-Seq data with a reference genome. In this case, RNA-Seq data was analyzed by series software, such as the Genome Analysis Toolkit (GATK) [15] or SAMtools [16], TopHat [17], and Cufflinks [18], to generate three inputs aimed at the construction of a customized database. The three inputs included a Variant Call Format (VCF) file containing single nucleotide variants (SNVs) and INDELs generated either by the GATK or SAMtools, a bed format file containing the junction information produced by TopHat, and a GTF format file containing novel transcripts reconstructed by Cufflinks. The other one is constructed from the analysis of RNA-Seq data without a reference genome. In this case, the transcript sequences were *de novo* assembled using software such as Trinity [19]. It is noted that the data format is important for the construction of a customized database, while the same data format, regardless of which software is used, is acceptable for PGA processing. To assist the construction of such a database with guidance from a reference genome, numerous pieces of genome annotation information, such as genome element region boundaries and protein coding sequences, were required, which were downloaded from Ensembl or the University of California, Santa Cruz (UCSC) table browser using the methods modified from customProDB. The functions and their uses for downloading this annotation information can be found in the user’s manual of PGA package. As for VCF and bed format files, customProDB could generate the RNA-Seq variants caused by SNVs, INDELs, and splice alternatives to the corresponding peptides. As for the GTF format file, the new transcripts were converted to the corresponding peptides based on three-frame translation with the strand information or six-frame translation without the strand information. Optionally, the new transcripts could be converted to peptides based on the longest open reading frame (ORF) in all reading frames. A customized proteomic database was therefore constructed, which contained all the canonical proteins, the potential novel peptides derived from RNA-Seq data, and their corresponding reverse sequences. All the proteins and peptides are in FASTA format and the FASTA headers for potential novel peptides are prefixed with “VAR” to distinguish them from the reference proteins. In general, a FASTA format file containing the *de novo* assembled transcript sequences that are achieved from the RNA-Seq analysis software, such as Trinity, but not from PGA, can be taken as input into PGA for proteomic database construction. As for this kind of database construction, the annotation information from Ensembl or UCSC is not required, and the transcript sequences can be translated to protein sequences by three-frame or six-frame translation or based on the longest ORF in all reading frames.

### MS/MS data searching

X!Tandem [20] is a well-accepted and open-source search engine, and was taken as the default database searching method in PGA. In the workflow of PGA, the R package rTANDEM [21], an R encapsulation of X!Tandem, was automatically used to search the customized proteomic database against MS/MS spectra. It can take the different MS/MS data formats as input in database searching, such as DTA, PKL, or MGF. Alternatively, search results with a dat format from MASCOT [22] or mzIdentML [23] format from MS-GF+ [24], MyriMatch [25], OMSSA [26] (converting OMSSA result to mzIdentML by mzidLibrary [27]), and IPeak [28, 29] were also accepted by PGA.

### Post-processing

X!Tandem Parser [30] was utilized to extract information of the peptide spectrum matches (PSMs) from the rTANDEM results. For taking the dat format file from MASCOT or the mzIdentML format file as input for the result of MS/MS data searching, MascotDatfile [31] or jmzIdentML [32] was used to extract this information, respectively. Taking into consideration the potential high false discovery rate (FDR) risk for novel peptide identification based on the customized proteomic database, which was constructed from the RNA-Seq data analysis with guidance from a reference genome, a so-called separate FDR estimation approach, proposed by Karpova et al. [33] for these identifications, was employed in PGA. The customized proteomics database contained the information regarding the RNA variants in the reference genome and the novel transcripts not annotated previously. If an identified peptide could not be mapped to the reference protein database, it was defined as a novel peptide. The FDR for novel peptides was estimated according to the following equation:

$$ FD{R}_n=\frac{D^{+}*\frac{D_n}{D}}{T_n^{+}} $$

where D^+^ is the number of identified decoy peptides with scores above the score threshold, T_n_^+^ is the number of identified novel peptides in the target database above the score threshold, D_n_ is the number of identified decoy novel peptides, and D is the total number of identified decoy peptides. D_n_/D is an approximation for the fraction of novel sequences in the search space. After PSM filtration based on a specified FDR threshold (commonly 1 %), the identified canonical peptide sequences were assembled into a set of confident proteins using the Occam’s razor approach [34], which provided a minimal list of proteins sufficient to explain all the identified canonical peptides. Finally, the two tab-delimited text files containing the identification results of peptides and proteins were exported. In addition, for each spectrum matched to a novel peptide, a file containing the annotated spectrum was also exported for a visualized quality check of the PSM. If an identified novel peptide was uniquely mapped to the amino acid sequences derived from the RNA variants, it was called a peptide variant in an existing gene. If an identified novel peptide was uniquely mapped to the amino acid sequences derived from the transcript never matched with the annotated gene, it was termed as the product of a novel gene.

### Generation of the HTML-based report

Using the R package Nozzle [35], PGA outputted an HTML-based interactive report, which contained summary plots and tables, annotated spectra, and identification information of novel peptides and canonical peptides.

## Results and discussion

PGA utility was evaluated using a published data set, in which RNA-Seq and proteomic data were collected from the Jurkat cell line in parallel [36]. The RNA-Seq data were downloaded from NCBI’s Gene Expression Omnibus (GEO) repository with the accession number GSE45428, and the MS/MS data were downloaded from the PeptideAtlas repository [37] with the accession number PASS00215. The detailed processing steps for the data are described in the Additional file 1. Two workflows were evaluated. The first one was that the protein identification was based on the customized proteomics database derived from the RNA-Seq data analysis with reference genome guidance. The second one was that the protein identification was based on the customized database derived from *de novo* transcriptome assembly from RNA-Seq data without reference genome guidance by Trinity.

**Fig. 2:**
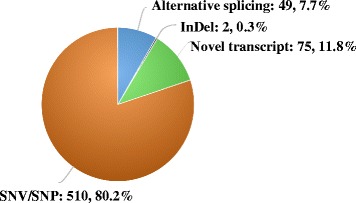
A pie diagram illustrating the results of novel peptide identification

**Fig. 3:**
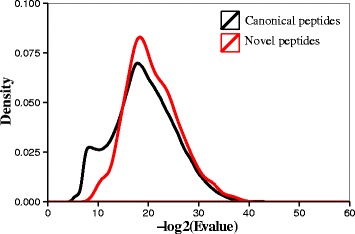
Search score distribution of novel and canonical peptides. The Evalue was an expectation value to evaluate the PSM confidence, and in this study, it was directly obtained from the search results of MASCOT. The greater the value of -log2(Evalue), the greater the confidence in the identifications

**Fig. 4:**
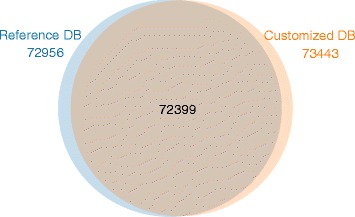
The overlap of peptides identified by searching the customized proteomic database and the reference database

With regards to the first workflow with reference genome guidance, the FDR threshold for identification of the canonical and novel peptides was set at 1 %. As shown in Fig. 2, in total, 636 novel peptides were identified by PGA, including 510 SAP peptides, 2 INDEL peptides, 49 splice junction peptides and 75 novel transcript-derived peptides. The distribution curves of PSM scores (−log2[Evalue]) illustrated in Fig. 3 revealed that the curve peak for novel peptides was basically close to that for the peptides mapped to the reference proteome, suggesting that the identification quality of novel peptides was acceptable. For most users, the HTML-based report automatically generated was fully informative and easily understandable. The report on the data set could be found in http://wenbostar.github.io/PGA/. In addition, as shown in Fig. 4, the number of peptides that were identified (73,443 peptides) based on searching the customized proteomics database was slightly higher than the number of peptides obtained based on searching the reference database (72,956 peptides).

**Fig. 5:**
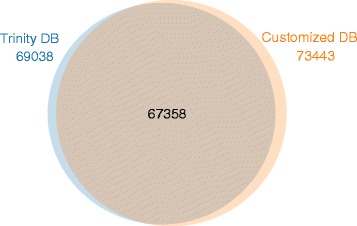
The overlap of peptides identified by the two workflows

In the absence of an organism genome, protein identification and quantification based on an MS approach were difficult to carry out due to the lack of corresponding gene sequences. In this case, the proteomic database derived from *de novo* assembly with RNA-Seq would be useful for MS/MS data searching. To test this postulation, the RNA-Seq data from the human Jurkat cell line were analyzed by Trinity as well, and the *de novo* assembly database was used for MS/MS searching. As the human proteome is arguably the best annotated of any species, it is possible to make a direct comparison of the results obtained with and without the use of a reference genome. As shown in Table 1, with the reads input to Trinity increasing (from ~5.6 M to ~ 81.9 M), the reconstructed transcripts (>200 bp) were proportionally augmented from 56,809 to 305,653, whereas the peptides identified appeared to be independent from the reads input, which reached a plateau (~69,000) once the reads were ~29 M or more. This implied that there is a threshold for the reads of RNA-Seq for peptide identification, whereas expansion of the data size in reads is not always beneficial to MS/MS search [38, 39]. Furthermore, we also compared the results from the two different workflows. As indicated in Fig. 5, about 91.71 % (67,358) of the identified peptides in the first workflow overlapped with that identified by the second workflow, suggesting that the identification results from the two workflows were comparable and each one could provide a small portion, approximately 10 %, of the compensative information.

**Table 1:** Identified transcripts and peptides at different numbers of input reads for Trinity

No. of reads	No. of transcripts (>200 bp)	No. of identified peptides (FDR <= 1 %)
5,602,829	56,809	65,841
12,568,426	99,797	68,473
28,886,097	174,587	69,038
47,136,664	233,256	68,900
81,871,805	305,653	68,236

## Conclusions

Using RNA-Seq data to enhance MS analysis is a promising strategy to discover novel peptides and to improve the sensitivity of peptide identification. The main bottleneck for widespread application of this strategy is lack of easily used software. We provided a novel end-to-end solution to this problem by introducing a complete pipeline in the Bioconductor environment. This software was evaluated in a data set of the RNA-Seq and proteomic data collected in a human cell line in parallel. Through construction of a customized proteomics database derived from RNA-Seq, PGA was demonstrated as a feasible program for discovering novel peptides arising from genetic variation, alternative splice forms, and novel coding genes.

## Availability and requirements

GPL-2 licensed and available in the Bioconductor framework.

- **Project name**: PGA software.
- **Project home page**: http://bioconductor.org/packages/PGA/.
- **Operating system(s)**: Linux, Mac OSX, Windows.
- **Programming language**: R, JAVA.
- **Other requirements**: None.
- **License**: GPL-2.
- **Any restrictions to use by non-academics**: GPL-2.

## Abbreviations

FDR, false discovery rate; GATK, the Genome Analysis Toolkit; MS/MS, Tandem mass spectrometry; PSM, peptide-spectrum match; SAP, single amino acid polymorphism; SNV, single nucleotide variants; VCF, Variant Call Format

## Additional file

Additional file 1: Supporting methods. (DOCX 27 kb)
