# Supplementary material for: PGA: an R/Bioconductor package for identification of novel peptides using a customized database derived from RNA-Seq
Source: BMC Bioinformatics. 2016 Jun 17;17:244. doi: 10.1186/s12859-016-1133-3 (PMC4912784; doi:10.1186/s12859-016-1133-3)
Supplement: Additional file 1: — Supporting methods. (DOCX 27 kb) [file 12859_2016_1133_MOESM1_ESM.docx]

# Supporting Information

PGA: an R/Bioconductor package for identification of novel peptides using a customized database derived from RNA-Seq

Bo Wen^1^#, Shaohang Xu^1^#, Ruo Zhou^1^, Bing Zhang^3^, Xiaojing Wang^3^, Xin Liu^1^, Xun Xu^1^ and Siqi Liu^1,2^*

^1^BGI-Shenzhen, Shenzhen, 518083, China.

^2^Beijing Institute of Genomics, Chinese Academy of Sciences, Beijing, 100101, China.

^3^Department of Biomedical Informatics, Vanderbilt University School of Medicine, Nashville, TN 37232, USA

# RNA-Seq data analysis

RNA-Seq data in FASTQ format was downloaded from NCBI’s Gene Expression Omnibus (GEO) repository with the accession number GSE45428. The sequencing data were first processed by filtering adaptor sequences and removing low-quality reads using SOAPnuke (version 1.5.2, <http://soap.genomics.org.cn/>) developed by BGI, and clean reads were generated. All clean reads were aligned to the Ensembl human genome (release GRCh37.75) using software TopHat (version 2.0.12) [[1](#_ENREF_1)] with parameter “--b2-very-sensitive,--segment-mismatches 2, and -r 150” to generate a sorted BAM file and a junction file. The mapped reads in the BAM file were then assembled into transcripts using Cufflinks (version 2.2.1) [[2](#_ENREF_2)] with the reference annotation file of human (release GRCh37.75), and finally a file named “transcripts.gtf” was generated. As for the SNPs and INDELs, we used the samtools mpileup command to calculate the genotype likelihoods supported by the aligned reads [[3](#_ENREF_3)]. And then the bcftools call command used the genotype likelihoods generated from the previous step to call SNPs and INDELs, and outputs all identified variants in the variant call format (VCF) [[4](#_ENREF_4)]. The annotation information was download from Ensembl by using the function “PrepareAnnotationEnsembl2” in PGA and six files were generated (1. exon_anno.RData”: exon annotation information, 2. procodingseq.RData: protein coding sequence (Nucleic acid sequence), 3. ids.RData: gene/transcript/protein id mapping information, 4. proseq.RData: protein coding sequence (Amino acid sequence), 5. splicemax.RData: annotated exon splice information, 6. txdb.sqlite: transcript database). The three files generated above together with the annotation information were used to construct the customized proteomic database by using the function “dbCreator” in PGA and a FASTA format database file was generated. Detailed usages about these functions can be found in the user’s manual of PGA. For the de novo transcriptome assembly, the Trinity (version trinityrnaseq_r20121005) [[5](#_ENREF_5)] software was used with the following parameters: --seqType fq --min_contig_length 100 --min_kmer_cov 2 --bfly_opts "-V 5 --edge-thr=0.05 --stderr" --group_pairs_distance 200. The transcript sequences less than 200 bp were removed. The proteomic database was constructed by using the function “createProDB4DenovoRNASeq” from PGA based on the assembled transcripts with translating the longest open reading frame (ORF) for each transcript to protein sequence.

# MS/MS data analysis

The MS/MS data was downloaded from the PeptideAtlas repository [[6](#_ENREF_6)] with the accession number PASS00215. The MS/MS spectra were searched by Mascot (v2.3.02, Matrix Science) against the customized proteomic database and the reference database, respectively. Mascot parameters were set as follows: two maximum missed cleavage of trypsin; fixed modifications including Carbamidomethyl (C); variable modifications consisting of Oxidation (M); 10 ppm of peptide mass tolerance; 0.05 Da of fragment mass tolerance. The Mascot result was then processed by MascotPercolator V2.12 [[7](#_ENREF_7), [8](#_ENREF_8)] with a new Mascot .dat file generated and finally processed by PGA.

# REFERENCES

1. Trapnell C, Pachter L, Salzberg SL: **TopHat: discovering splice junctions with RNA-Seq**. *Bioinformatics* 2009, **25**(9):1105-1111.

2. Trapnell C, Williams BA, Pertea G, Mortazavi A, Kwan G, van Baren MJ, Salzberg SL, Wold BJ, Pachter L: **Transcript assembly and quantification by RNA-Seq reveals unannotated transcripts and isoform switching during cell differentiation**. *Nat Biotechnol* 2010, **28**(5):511-515.

3. Li H, Handsaker B, Wysoker A, Fennell T, Ruan J, Homer N, Marth G, Abecasis G, Durbin R, Genome Project Data Processing S: **The Sequence Alignment/Map format and SAMtools**. *Bioinformatics* 2009, **25**(16):2078-2079.

4. Li H: **A statistical framework for SNP calling, mutation discovery, association mapping and population genetical parameter estimation from sequencing data**. *Bioinformatics* 2011, **27**(21):2987-2993.

5. Grabherr MG, Haas BJ, Yassour M, Levin JZ, Thompson DA, Amit I, Adiconis X, Fan L, Raychowdhury R, Zeng Q *et al*: **Full-length transcriptome assembly from RNA-Seq data without a reference genome**. *Nat Biotechnol* 2011, **29**(7):644-652.

6. Desiere F, Deutsch EW, King NL, Nesvizhskii AI, Mallick P, Eng J, Chen S, Eddes J, Loevenich SN, Aebersold R: **The peptideatlas project**. *Nucleic acids research* 2006, **34**(suppl 1):D655-D658.

7. Brosch M, Yu L, Hubbard T, Choudhary J: **Accurate and sensitive peptide identification with Mascot Percolator**. *J Proteome Res* 2009, **8**(6):3176-3181.

8. Kall L, Canterbury JD, Weston J, Noble WS, MacCoss MJ: **Semi-supervised learning for peptide identification from shotgun proteomics datasets**. *Nat Methods* 2007, **4**(11):923-925.
